# Simple adjustment of the sequence weight algorithm remarkably enhances PSI-BLAST performance

**DOI:** 10.1186/s12859-017-1686-9

**Published:** 2017-06-02

**Authors:** Toshiyuki Oda, Kyungtaek Lim, Kentaro Tomii

**Affiliations:** 1 Artificial Intelligence Research Center, National Institute of Advanced Industrial Science and Technology (AIST), 2-4-7 Aomi, Koto-ku, Tokyo, 135-0064 Japan; 2 Biotechnology Research Institute for Drug Discovery, National Institute of Advanced Industrial Science and Technology (AIST), 2-4-7 Aomi, Koto-ku, Tokyo, 135-0064 Japan

**Keywords:** PSI-BLAST, Sequence similarity search, Sequence weighting, Position-specific scoring matrix

## Abstract

**Background:**

PSI-BLAST, an extremely popular tool for sequence similarity search, features the utilization of Position-Specific Scoring Matrix (PSSM) constructed from a multiple sequence alignment (MSA). PSSM allows the detection of more distant homologs than a general amino acid substitution matrix does. An accurate estimation of the weights for sequences in an MSA is crucially important for PSSM construction. PSI-BLAST divides a given MSA into multiple blocks, for which sequence weights are calculated. When the block width becomes very narrow, the sequence weight calculation can be odd.

**Results:**

We demonstrate that PSI-BLAST indeed generates a significant fraction of blocks having width less than 5, thereby degrading the PSI-BLAST performance. We revised the code of PSI-BLAST to prevent the blocks from being narrower than a given minimum block width (MBW). We designate the modified application of PSI-BLAST as PSI-BLASTexB. When MBW is 25, PSI-BLASTexB notably outperforms PSI-BLAST consistently for three independent benchmark sets. The performance boost is even more drastic when an MSA, instead of a sequence, is used as a query.

**Conclusions:**

Our results demonstrate that the generation of narrow-width blocks during the sequence weight calculation is a critically important factor that restricts the PSI-BLAST search performance. By preventing narrow blocks, PSI-BLASTexB upgrades the PSI-BLAST performance remarkably. Binaries and source codes of PSI-BLASTexB (MBW = 25) are available at https://github.com/kyungtaekLIM/PSI-BLASTexB.

**Electronic supplementary material:**

The online version of this article (doi:10.1186/s12859-017-1686-9) contains supplementary material, which is available to authorized users.

## Background

Sequence similarity search is an initial choice for structural and functional inference of unknown biological sequences, for which BLAST [1] is widely used. BLAST uses an amino acid substitution matrix such as BLOSUM62 [2] to score similarities between amino acid pairs. Starting from the original BLAST, it has evolved in several aspects, such as gap treatment [3] and composition-based adjustment [4]. Using an iterative search, BLAST (precisely, PSI-BLAST [3]) can employ patterns of amino acids varying among homologs and among positions within homologs. It can therefore detect more distant homologs than the original BLAST does.

The multiple sequence alignment (MSA) of closely related homologous sequences detected by BLAST is expected to contain such homolog-specific and position-specific information. An MSA can be transformed into a position-specific scoring matrix (PSSM), which is a more sophisticated model for sequence similarity search than the substitution matrix because scores for amino acids are modeled for individual positions. Iterative search methods including PSI-BLAST [3] construct a PSSM from an MSA obtained from the previous search. Then such methods use the PSSM for another similarity search. It has been demonstrated that much more distant homologs can be detected by iterating these steps. Because of its usefulness and availability, many modifications have been proposed since PSI-BLAST was first published, including introduction of composition-based statistics, optimizing cache utilization, and revising pseudo-count strategy [4–6]. Overcoming the problem of “homologous over-extension (HOE)” also improves the PSI-BLAST accuracy [7, 8]. In this study, we describe that PSI-BLAST can be improved further by slightly changing the sequence weighting method.

Because sequences in public databases are highly biased into organisms that are medically and commercially important, and because they are easy to culture, it is crucially important to adjust amino acid observations in the MSA of homologous sequences before PSSM calculation. Sequence weight is a straightforward way for attaining such adjustment, where a sequence with more closely related counterparts in the MSA should be assigned a smaller weight. PSI-BLAST calculates the position-specific sequence weight (PSSW) using a procedure derived from the formula proposed [9] as

$$ {W}_i = {\displaystyle {\sum}_{j=1}^l}1/\left({r}_j*{n}_{aj}* l\right) $$

where *Wi* stands for the weight of *i*th sequence in a MSA, *r*_*j*_ denotes the number of unique amino acids found at the position *j*, *l* signifies the length of the alignment, and *n*_*aj*_ represents the number of amino acids *a* found at *j*. After sequences are weighted, the probability of *a* at *j* (*P*_*aj*_) is calculated as

$$ {P}_{aj}={\sum}_{i=1}^n{W}_i\ast t, $$

$$ t = \left\{\begin{array}{c}\hfill 1\  if\ {u}_{ij}= a\hfill \\ {}\hfill 0\  if\ {u}_{ij}\ne a\hfill \end{array}\right., $$

where *u*_*ij*_ stands for the amino acid at *j* in the *i*th sequence, and *n* signifies the number of sequences in MSA.

This formula lacks the consideration of gaps. Simply put, gaps (including N-terminal and C-terminal gaps) can be treated as the 21st amino acid. An important problem of this approach is that the weights of gappy sequences in a gappy MSA will be underestimated. One can avoid this problem by considering an MSA subregion with few or no gaps for PSSW calculation. This is expected to be advantageous for dealing with MSAs constructed from local alignments that are likely to include many gaps. PSI-BLAST defines such blocks for individual positions. PSI-BLAST first selects a subset of sequences (a reduced MSA) in an MSA, such that no gap is included at a position of interest *j*. PSI-BLAST then collects starting and ending positions of all pairwise alignments between query and subjects in the reduced MSA to define the boundary of the block as the starting and ending positions closest to *j* [3]. This approach also has an important limitation: The block width can be extremely narrow, failing to reflect actual evolutionary information.

This study demonstrates that such narrow blocks are created during the PSSM construction of PSI-BLAST, which gives rise to inaccurate calculation of PSSW and PSSM, and which thereby drastically hampers the homology detection performance. We propose a simple method for better PSSW calculation, which boosts the PSI-BLAST performance.

## Implementation

### Narrow blocks result in wrong sequence weight calculation

**Fig. 1:**
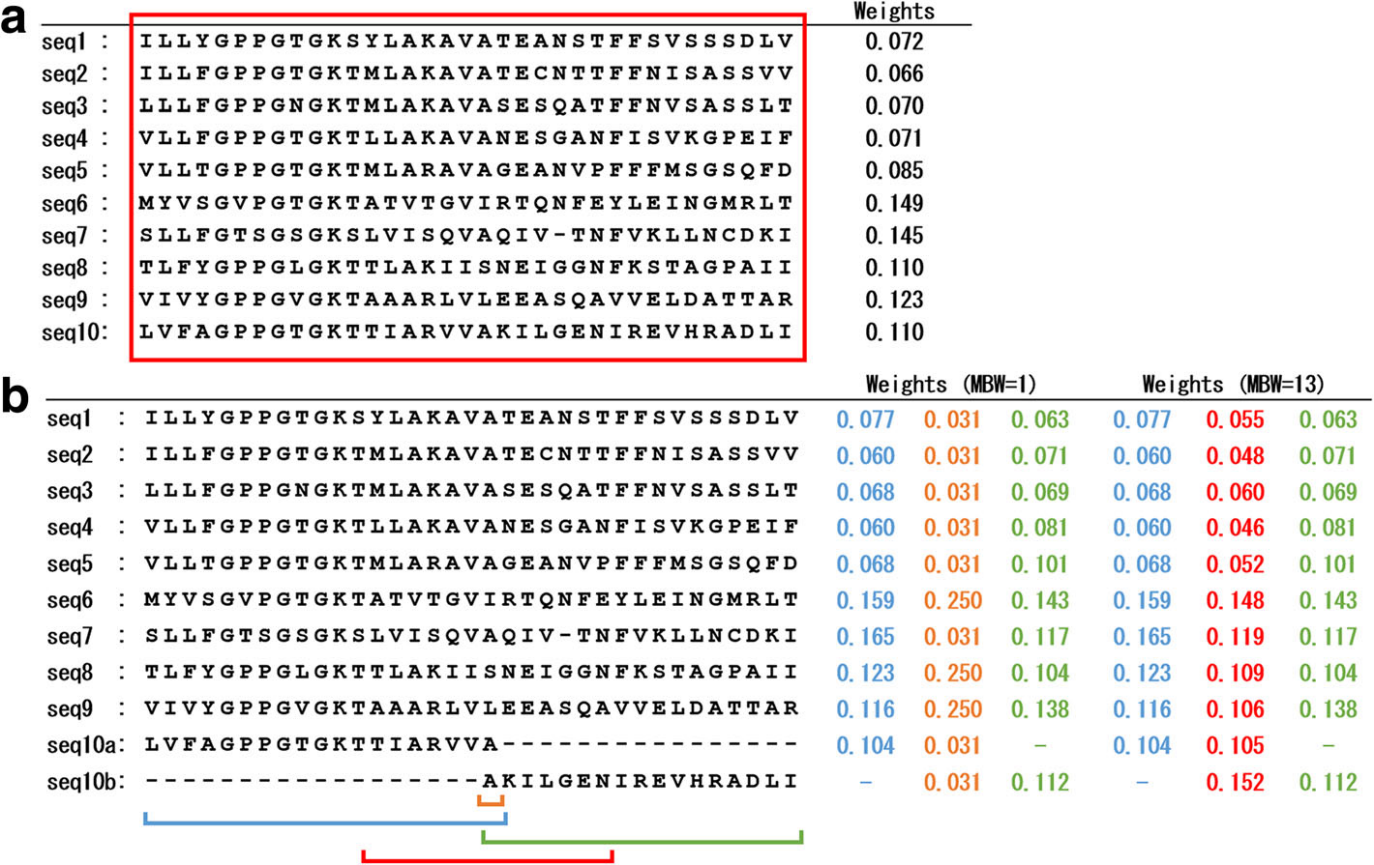
Examples showing the sequence weight calculation of PSI-BLAST and PSI-BLASTexB. **a** Sequence weights (shown on the right side) of all positions in the MSA were calculated from a single block covering the whole alignment. **b** PSI-BLAST divided the MSA into three blocks (blue, orange, and green) and calculated sequence weights for each block. Sequence weights calculated from the blocks are shown on the right side with the same color. For the orange block that is one aa long, PSI-BLASTexB extends the block such that the block width becomes MBW (red block). Weights calculated from the red block are also shown. See *Methods* for detailed procedures. “seq7” has no amino acid at position 23. For that reason, the sequence weights of other sequences are calculated ignoring “seq7” at the position

To exemplify the effect of narrow blocks, we show two artificial MSAs presented in Fig. 1. The MSA in Fig. 1a (MSA-A) is a subset of AAA ATPase MSA in the Pfam database [10]. The MSA in Fig. 1b (MSA-B) is identical to MSA-A except for 10th and 11th sequences, which were derived from the 10th sequence in MSA-A by dividing it into two pieces with an overlap at position 19. The two MSAs were converted to PSSMs (Additional files 1 and 2, respectively) by PSI-BLAST search against a dummy database with “-in_msa”, “-num_iterations 1” and ‘-out_ascii_pssm’ options.

We checked the inner variables of PSI-BLAST to mark blocks on MSA-A and MSA-B (Fig. 1). A block that covers the whole MSA was used for all positions in MSA-A because it lacks gaps, whereas three blocks were generated for MSA-B, where the block width (*l*) at position 19 is one (Fig. 1b, orange block). At position 19, the weights of the sequences not only of seq10a and seq10b but also of seq1-9 in MSA-B deviate drastically from those in MSA-A. Consequently, at position 19 of MSA-B, the weighted percentage of alanine, leucine, isoleucine, and serine were equally 25 (Additional file 2). Because when *l* is one, the number of sequences which have *a* at *j* is *n*_*aj*_. The weighted probabilities of amino acids are 1/(*r*_j *_ *n*_*aj*_)* *n*_*aj*_ = 1/*r*_*j*_. In MSA-A, the weighted percentage of those 4 amino acids were 62, 15, 12, and 11, respectively (Additional file 1), demonstrating the limitation of PSI-BLAST PSSW calculation when the block width is tiny.

### Block extended PSI-BLAST (PSI-BLASTexB)

A simple and direct solution of this problem is to prevent block widths from being narrower than a certain width by exceptionally allowing gaps in the blocks. These gaps might cause the underestimation of gappy sequences in an alignment as discussed above, which however would certainly be a better estimation than the weights calculated for blocks having width of several residues.

The PSI-BLAST source code in the BLAST+ package [11] was downloaded from the BLAST FTP site (ftp://ftp.ncbi.nlm.nih.gov/blast/executables/blast+/2.5.0/). We revised the PSI-BLAST code and added lines after line 1415 of ncbi-blast-2.5.0 + −src/c++/src/algo/blast/core/blast_psi_priv.c, as shown below.



It implements the minimum block width (MBW), which is “1” in the original code. Blocks with widths < MBW are extended front and rear by MBW-1 until the termini of the MSA. For example, when MBW is 13, the deviated weights of MSA-B (Fig. 1b, red block) became similar to the weights of MSA-A (Fig. 1a). The resulting PSSM of MSA-B with MBW13 is provided as Additional file 3. The source code was configured with “--with-bin-release” and “--with-ncbi-public” options and compiled by the make command with no options. We designate the modified PSI-BLAST as PSI-BLASTexB.

### Benchmark dataset

The search performance was compared using SCOP20_training, SCOP20_validation and CATH20-SCOP datasets, as established in our previous study [12]. The SCOP20_training and SCOP20_validation datasets were derived from the non-redundant set of 7074 proteins (SCOP20), which was provided by the ASTRAL compendium [13]. The 7074 sequences were divided into two groups for parameter optimization (SCOP20_training) and performance evaluation (SCOP20_validation). CATH20-SCOP dataset was derived from the CATH database [14] excluding sequences in the SCOP database. The sequences in the datasets were filtered so that the sequences did not have > 20% mutual sequence identity. Finally, our dataset included respectively 3537, 3537, and 1754 sequences. All datasets are available from http://csas.cbrc.jp/Ssearch/benchmark/.

### PSSM construction

PSSMs for individual sequences in the benchmark datasets were constructed using PSI-BLAST and PSI-BLASTexB against the Uniref50 dataset (Release 2015_10) [15] downloaded from the UniProt FTP site (ftp://ftp.uniprot.org/pub/databases/uniprot/uniref/uniref50/). In this study, PSSMs for iteration X were generated using the following command:

psiblast -query < QUERY > −db < UNIREF50 DB > −out_pssm < PSSM PATH > −num_iterations < X > −num_alignments 1000000.

We also extracted an MSA consisting of hits from a PSI-BLAST search with “-num_interation 1” option, and used the MSA directly to another search against Uniref50 using the “-in_msa” option, which is an alternative method of running an iterative PSI-BLAST search with an MSA instead of a query (“-query”) or checkpoint PSSM (“-in_pssm”).

### Performance evaluation

Similarity searches were conducted respectively against benchmark datasets using the constructed PSSMs and MSAs as queries using the “-in_pssm” and “-in_msa” options. We followed the rule set proposed by Julian Gough (http://www.supfam.org/SUPERFAMILY/ruleset.html) [16] to define true positive (TP) and false positive (FP) hits at the superfamily level. Superfamily definitions of the rule set differ from the original ones of SCOP. The rule set also excludes hits with a potential homologous relation from FPs.

To evaluate the performance, we introduced a receiver operating characteristic (ROC) curve plot, which has been used widely for performance evaluation [17, 18]. Hits from all queries were pooled and ranked by their E-values. Then TP and FP hits until various E-value thresholds were counted and shown, with weighting of the TP and FP counts by 1/(number of all TPs in the dataset) for each query.

We also calculated the ROC5 score for hits with E-values less than 1.0, which indicates the search performance of individual queries using the following equation:

$$ ROC5=\frac{1}{5 T}{\sum}_{i=1}^5{t}_i. $$

Therein, *T* signifies the total TP count; *t*_*i*_ denotes the TP count until the *i*-th FP appears [19].

## Results and Discussion

**Fig. 2:**
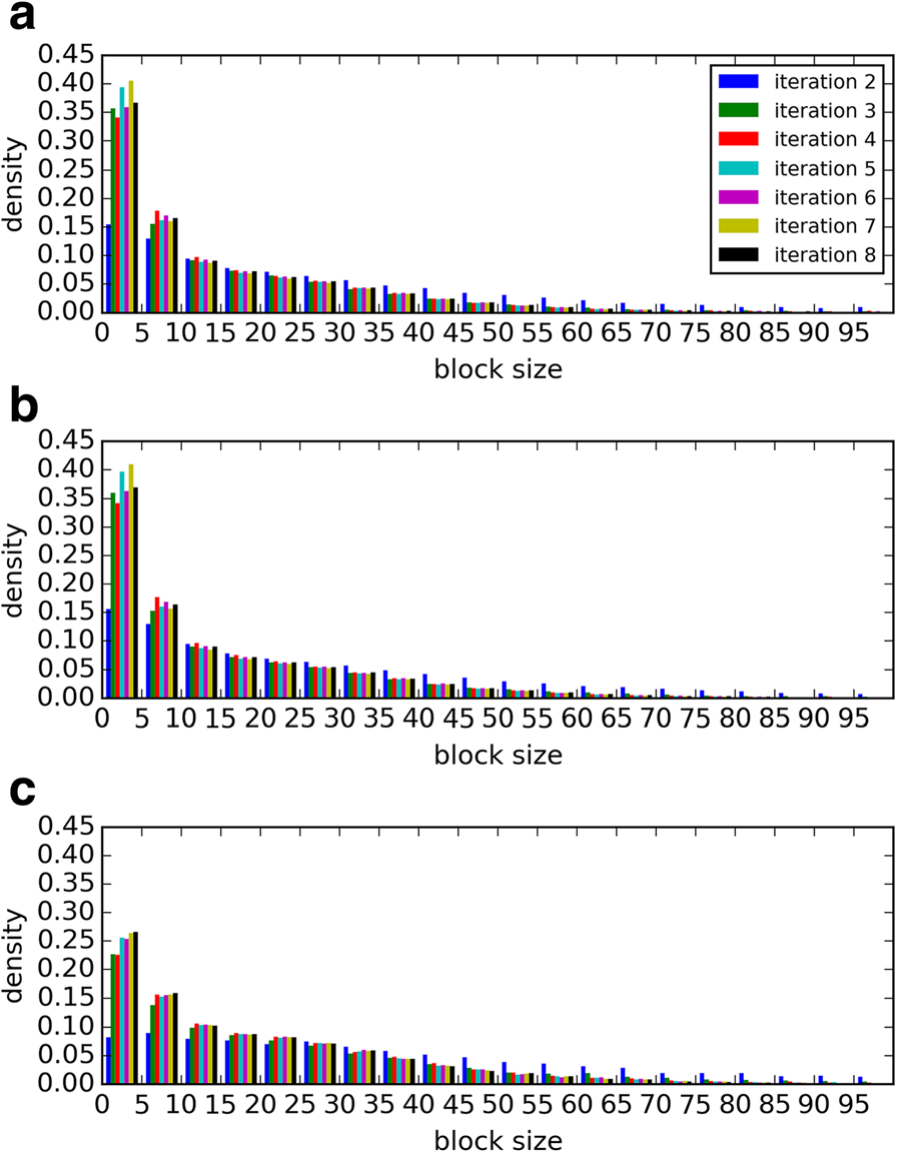
Distributions of block widths used for PSSW calculation with varying numbers of iterations. Results of searches against SCOP20_training, SCOP20_validation, and CATH20-SCOP are presented, respectively, in (**a**) (**b**) and (**c**). Searches that converged before the eighth iteration were not used. Numbers of sequences (and blocks) used in (**a**) (**b**) and (**c**) are, respectively, 2502 (468286), 2473 (459375), and 1009 (135176). Numbers of searches that had not converged before each iteration are provided in Additional file 4: Table S1

We first investigated how many narrow-width blocks, which are potentially causing the problem of sensitivity reduction, are generated during PSI-BLAST searches. We therefore measured the distribution of block widths used for individual query positions by PSI-BLAST at the second to eighth iterations for three independent benchmark sets (Fig. 2). About 35%, 35%, and 25% of the blocks had widths of less than 5 amino acids (aa) at the eighth iteration of SCOP20_training, SCOP20_validation, and CATH20-SCOP datasets, respectively. This fact demonstrates that PSI-BLAST produces the narrow-width blocks constantly.

**Fig. 3:**
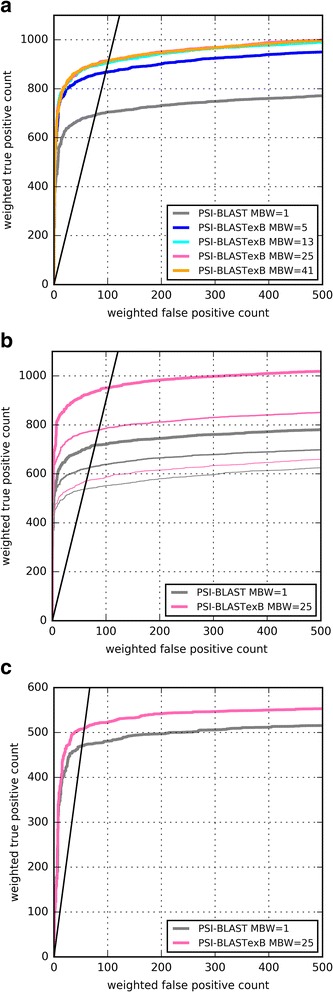
ROC curves of PSI-BLAST and PSI-BLASTexB. **a** ROC curves of PSI-BLAST (MBW = 1) and PSI-BLASTexB (MBW = 5, 13, 25, or 41) at the fifth iteration against SCOP20_training. **b** ROC curves among searches with different numbers of iterations against SCOP20_validation. Narrow, normal, and thick lines respectively show the second, third, and fifth iterations. **c** ROC curves of PSI-BLAST and PSI-BLASTexB at the fifth iteration against CATH20-SCOP. Black lines represent FDR of 10%

Using the SCOP20_training dataset, we analyzed the PSI-BLASTexB performance with varying MBW values (5, 13, 25, and 41) at the fifth iteration. PSI-BLAST corresponds to PSI-BLASTexB with the MBW of one. As Fig. 3a shows, the performance of PSI-BLASTexB is much higher than that of PSI-BLAST across all MBW values. The performances are almost identical when MBW values are 13, 25, and 41, and are slightly low when MBW is 5, which suggests that 5 aa long blocks are insufficient to calculate the correct PSSW. The weighted TP count was highest when MBW was 25 at the false discovery rate (FDR) of 10%. Therefore, we use the value as the default in the following experiments.

The performance improvement was also clear for SCOP20_validation and CATH20-SCOP (Figs. 3b and c). However, the performance improvement for CATH20-SCOP was slight compared with those of SCOP20_training and SCOP20_validation. That result is consistent with the result of the distributions of block widths. The fractions of narrow-width blocks in CATH20-SCOP are smaller than those of SCOP20_training and SCOP20_validation (Fig. 2), which is expected because our new method would be of no use if few narrow-width blocks existed.

**Fig. 4:**
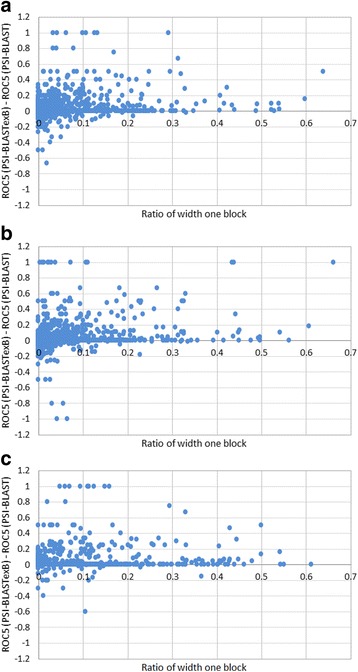
Relations between the ROC5 score improvement and the fraction of narrow blocks. The X-axis shows (number of one aa long blocks during PSSM construction)/(length of the query). The Y-axis shows the ROC5 score of PSI-BLASTexB replaced by that of PSI-BLAST. Each dot represents the result of a single query. The results of queries which have only one TP hit (self-hit) were ignored. Results of SCOP20_training (2752 queries), SCOP20_validation (2752 queries), and CATH20-SCOP (858 queries) at the second iteration are presented respectively in A, B, and C

To observe the relation between performance improvement and the block extension for each query, the incremental ROC5 scores (ROC5 score by PSI-BLASTexB - ROC5 score by PSI-BLAST) are shown against the ratio of positions with one aa long blocks at the second iteration for each query (Fig. 4). When the ratio is larger than 0.1, in other words, when more than 10% of PSSM positions are derived from one aa long blocks, 92, 90, and 81 PSI-BLASTexB searches among 189, 195, and 196 achieve higher performance than PSI-BLAST searches. Only for 10, 11, and 9 cases are PSI-BLASTexB searches worse, respectively, than PSI-BLAST searches against SCOP20_training, SCOP20_validation, and CATH20-SCOP. In contrast, improvement of queries with the ratio less than 0.1 appears to be more random, although PSI-BLASTexB searches are also effective for many queries with the ratio less than 0.1. These results show how widening the widths of narrow blocks improves the search performance.

**Fig. 5:**
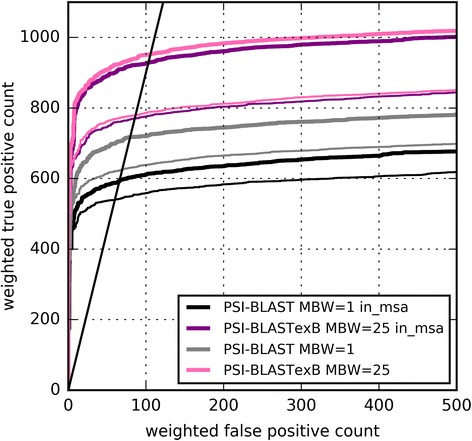
ROC curves with “-in_msa” option of PSI-BLAST and PSI-BLASTexB against SCOP20_validation. Thick and narrow lines respectively show ROC curves at the fifth and third iterations. The black straight line shows FDR of 10%

PSI-BLAST supports a search using an MSA as an input with “-in_msa” option. We constructed MSAs from the outputs of PSI-BLAST and PSI-BLASTexB to use them as queries for the next search (see *Methods* for details). As Fig. 5 shows, the performance of PSI-BLAST with “-in_msa” option is distinguishably lower than that of normal PSI-BLAST search with the corresponding number of iterations. From our understanding, when “-in_msa” is used, PSI-BLAST divides a sequence in an MSA into multiple pieces if large gaps exist within (10 aa in case of ver. 2.5.0). Therefore, more narrow-width blocks are generated with the “-in_msa” option. Block extension by PSI-BLASTexB effectively suppresses performance degradation using MSAs as queries (Fig. 5). Therefore, PSI-BLASTexB can facilitate the use of MSAs prepared in advance as queries, e.g. Pfam seed alignments [10], HMM-HMM alignments by HHblits [20], and progressive alignments by MAFFT [21] for distant homology detection.

**Fig. 6:**
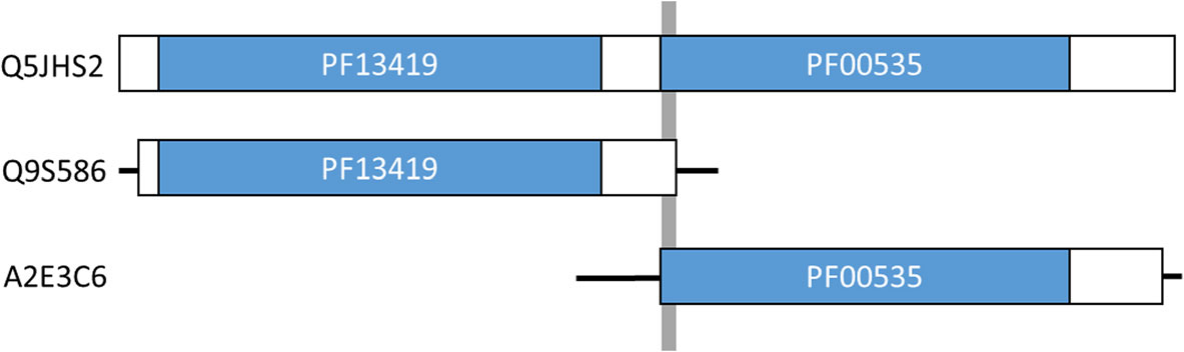
Schematic representation of narrow-width block generation by HOE. When we performed a PSI-BLAST search [22], at the NCBI website, of a protein sequence (UniProtKB [23] accession number: Q5JHS2, as a query) that contains two conserved domains (Pfam [10] IDs: PF13419 and PF00535) against the UniProtKB/Swiss-Prot database [24], we found that a hit (UniProtKB accession number: Q9S586) consisting of a single-domain protein (Pfam ID: PF13419) with HOE (*white* boxes) has an overlap with another hit (UniProtKB accession number: A2E3C6) matched only to the domain of PF00535, resulting in a 3 aa-long block (*gray* bar)

We presume that troubles of at least two types can be sources of narrow-width blocks in an MSA, although such blocks might also arise from other sources. One is an HOE [8] related problem. We present an example of this phenomenon in Fig. 6. When multiple conserved regions (often domains) exist in a query, narrow-width blocks are likely to be included in the resulting MSA attributable to overlaps between extended non-homologous residues flanking a conserved region and an adjacent conserved region. Mainly, this is a query-dependent problem. Some solutions have been proposed [7, 8]. The other is an issue of the sequence library. As shown in Fig. 1b, fragmented sequences in libraries can produce narrow-width blocks caused by their overlaps. Therefore, dividing queries such that each query has only one conserved region or removing fragmented sequences from the library should be workarounds to reduce the number of narrow-width blocks. However, the practical applications of these procedures might require further consideration (e.g., how to determine “conserved” and “fragmented”). Consequently, our simple adjustment of the sequence weight algorithm is a more practical way of handling narrow-width blocks in a MSA produced by PSI-BLAST.

## Conclusion

Because of sequence weighting scheme limitations, the PSI-BLAST performance has been penalized until now. We developed a customized PSI-BLAST, designated as PSI-BLASTexB, which solved such problems with extremely simple modification of the PSI-BLAST code. PSI-BLASTexB significantly outperformed PSI-BLAST. Therefore, it is expected to be useful not only for distant homology search, but also for many downstream methods that depend on PSI-BLAST with trivial effort.

## Additional files

Additional file 1: The ascii pssm file made from MSA-A using PSI-BLAST. (ASCII 5 kb)

Additional file 2: The ascii pssm file made from MSA-B using PSI-BLAST. (ASCII 5 kb)

Additional file 3: The ascii pssm file made from MSA-B using PSI-BLASTexB with setting minimum block width as 13. (ASCII 5 kb)

Additional file 4: Table S1. The number of searches which were not converged before each iteration of PSI-BLAST. (XLSX 9 kb)

## Abbreviations

AA: Amino acid
FDR: False discovery rate
FP: False positive
HOE: Homologous over-extension
MBW: Minimum block width
MSA: Multiple sequence alignment
PSSM: Position-specific scoring matrix
PSSW: Position-specific sequence weight
ROC: Receiver operating characteristic
TP: True positive

## References

[1] Altschul SF, Gish W, Miller W, Myers EW, Lipman DJ (1990). Basic local alignment search tool. J Mol Biol.

[2] Henikoff S, Henikoff JG (1992). Amino acid substitution matrices from protein blocks. Proc Natl Acad Sci U S A.

[3] Altschul SF, Madden TL, Schaffer AA, Zhang J, Zhang Z, Miller W, Lipman DJ (1997). Gapped BLAST and PSI-BLAST: a new generation of protein database search programs. Nucleic Acids Res.

[4] Schaffer AA, Aravind L, Madden TL, Shavirin S, Spouge JL, Wolf YI, Koonin EV, Altschul SF (2001). Improving the accuracy of PSI-BLAST protein database searches with composition-based statistics and other refinements. Nucleic Acids Res.

[5] Altschul SF, Gertz EM, Agarwala R, Schaffer AA, Yu YK (2009). PSI-BLAST pseudocounts and the minimum description length principle. Nucleic Acids Res.

[6] Aspnas M, Mattila K, Osowski K, Westerholm J (2010). Code optimization of the subroutine to remove near identical matches in the sequence database homology search tool PSI-BLAST. J Comput Biol.

[7] Li W, McWilliam H, Goujon M, Cowley A, Lopez R, Pearson WR (2012). PSI-Search: iterative HOE-reduced profile SSEARCH searching. Bioinformatics.

[8] Gonzalez MW, Pearson WR (2010). Homologous over-extension: a challenge for iterative similarity searches. Nucleic Acids Res.

[9] Henikoff S, Henikoff JG (1994). Position-based sequence weights. J Mol Biol.

[10] Finn RD, Coggill P, Eberhardt RY, Eddy SR, Mistry J, Mitchell AL, Potter SC, Punta M, Qureshi M, Sangrador-Vegas A (2016). The Pfam protein families database: towards a more sustainable future. Nucleic Acids Res.

[11] Camacho C, Coulouris G, Avagyan V, Ma N, Papadopoulos J, Bealer K, Madden TL (2009). BLAST+: architecture and applications. BMC Bioinformatics.

[12] Yamada K, Tomii K (2014). Revisiting amino acid substitution matrices for identifying distantly related proteins. Bioinformatics.

[13] Fox NK, Brenner SE, Chandonia JM (2014). SCOPe: Structural Classification of Proteins--extended, integrating SCOP and ASTRAL data and classification of new structures. Nucleic Acids Res.

[14] Sillitoe I, Lewis TE, Cuff A, Das S, Ashford P, Dawson NL, Furnham N, Laskowski RA, Lee D, Lees JG (2015). CATH: comprehensive structural and functional annotations for genome sequences. Nucleic Acids Res.

[15] Suzek BE, Wang Y, Huang H, McGarvey PB, Wu CH, UniProt C (2015). UniRef clusters: a comprehensive and scalable alternative for improving sequence similarity searches. Bioinformatics.

[16] Gough J, Karplus K, Hughey R, Chothia C (2001). Assignment of homology to genome sequences using a library of hidden Markov models that represent all proteins of known structure. J Mol Biol.

[17] Angermuller C, Biegert A, Soding J (2012). Discriminative modelling of context-specific amino acid substitution probabilities. Bioinformatics.

[18] Biegert A, Soding J (2009). Sequence context-specific profiles for homology searching. Proc Natl Acad Sci U S A.

[19] Gribskov M, Robinson NL (1996). Use of receiver operating characteristic (ROC) analysis to evaluate sequence matching. Comput Chem.

[20] Remmert M, Biegert A, Hauser A, Soding J (2012). HHblits: lightning-fast iterative protein sequence searching by HMM-HMM alignment. Nat Methods.

[21] Katoh K, Standley DM (2013). MAFFT multiple sequence alignment software version 7: improvements in performance and usability. Mol Biol Evol.

[22] Boratyn GM, Camacho C, Cooper PS, Coulouris G, Fong A, Ma N, Madden TL, Matten WT, McGinnis SD, Merezhuk Y (2013). BLAST: a more efficient report with usability improvements. Nucleic Acids Res.

[23] Pundir S, Martin MJ, O’Donovan C (2017). UniProt Protein Knowledgebase. Methods Mol Biol.

[24] Boutet E, Lieberherr D, Tognolli M, Schneider M, Bansal P, Bridge AJ, Poux S, Bougueleret L, Xenarios I (2016). UniProtKB/Swiss-Prot, the Manually Annotated Section of the UniProt KnowledgeBase: How to Use the Entry View. Methods Mol Biol.

